# Influence of Heat and Thermochemical Treatment Parameters on C75 Steel Fatigue Resistance

**DOI:** 10.3390/ma15155378

**Published:** 2022-08-04

**Authors:** Tudorache (Nistor) Iuliana, Cornel Samoila, Doru Ursutiu

**Affiliations:** 1 Transilvania University of Brașov, 500036 Brasov, Romania; 2 The Romanian Academy of Technical Sciences, 010071 Bucharest, Romania; 3 The Academy of Romanian Scientists, 050094 Bucharest, Romania

**Keywords:** fatigue resistance, computed tomography, microstructure, macro-structural analysis

## Abstract

The paper presents the results of the fatigue testing of heat-treated and thermochemically treated C75 steel with different process parameters in terms of working medium (gas, salt bath), temperature, and time. The experimental program aims to analyze the changes in microstructure under the influence of heat treatment and fatigue resistance. The relationships between the structural changes, the internal stress, and the heat-treated material’s mechanical and physical properties can determine the first nano cracks leading to rupture propagation. Based on the experimental values of this paper, we highlight the dependence between the nature of the cracks and the stress to which the specimen was subjected. The paper presents a brief introduction to the fatigue test and the experimental tests performed to determine the fatigue resistance characteristics, the macroscopic analysis of the material, and the crystallographic analysis. The results obtained allow a comparison between the fatigue limits of heat-treated and thermochemically treated C75 steel in gas and salt baths.

## 1. Introduction

Most materials used in industry are subject to repeated cyclic stresses. Although the intensity of the stress is usually below the fracture strength of the material, this leads to destruction over time. The influence of variable loads on the fatigue process is determined by the number and amplitude of variations in stress over the life of the structure [1]. The reference intensity for these cyclic stresses is fatigue resistance, and its determination is regulated by the SR ISO 1099 standard: “*Metallic Materials-Fatigue testing-Axial force-controlled method*” 2017 [2]. Unlike the concept of “*infinite-life design*”, which requires the classification of working stress within the limits of material elasticity, there is also the concept of “*safe-life design*”, that aims for the material to withstand several variable stress cycles without failure in the time in which it is used. However, this concept brings many complications in the approach to fatigue resistance calculations due to less reliable assumptions, so another concept was launched, namely, “*fail-safe design*”, i.e., design with controlled deterioration. This concept must be accompanied by an additional one, namely, “*safe by inspection*”, which assesses the level of risk through periodic inspections. This tolerance to defects allows fatigue cracks in the material until the loss of physical integrity. This concept assumes that there are methods for determining the occurrence of nano cracks, i.e., the beginning of the structure destruction. This research seeks to obtain the preliminary data necessary for designing a Barkhausen noise-based device that can show not only the occurrence of nano cracks but can also estimate the remaining life if failure has begun, using magnetic methods, and based on 1/f noise. From a mechanical point of view, the crack is a discontinuity of the material, or its crystalline structure, more precisely. Non-destructive testing methods have been developed so as not to change the material being inspected. All discontinuities may affect fatigue resistance whether they are generated during design, processing, or operation. There are indirect methods of consolidation for this; this paper presents types of heat and thermochemical treatments and analyzes their influence on fatigue resistance.

## 2. State of the Art

Fatigue research is more than 150 years old. The first known results of fatigue testing were published in 1837 by Albert [3]. A rather tragic accident took place on May 11, 1842, when a train derailed on the railway between Versailles and Paris after the leading locomotive broke an axle and the carriages behind caught fire. It was the first accident in France to cause 52 deaths. The accident was investigated by Smith [4], a step that led to the design of machine parts based on fatigue resistance. Many other accidents have caused ruptures in aircraft landing gear, resistance coating, or propeller blades [5]. There is extensive research on how heat or thermochemical treatment can improve fatigue resistance. The correct choice of heat treatment brings the microstructure and mechanical properties of steel to optimal values for fatigue resistance [6]. In [7], the initiation of fatigue cracks was evaluated on cylindrical samples that were subjected to fatigue testing with certain amplitude. A study on the initiation of fatigue cracks was performed by interrupting the cycles at a defined number and studying the surfaces using scanning electron microscopy. The researchers found that the cyclic deformation was located at the first interval. Other authors have tried to study the increase in fatigue resistance by using AHSS steels (advanced high-strength steels) subjected to different heat treatments. Increased fatigue resistance has been studied by using advanced high-strength steels with different heat treatments. Kwon et al. studied two types of steel, namely, conventional and modified steel, which hardened to 930 °C and had different tempering temperatures between 100 and 340 °C. Standard steels were tempered at 340 °C and modified steels at 100, 200, 250, 300, 340, and 400 °C. Following the experiments, the authors found that the specimen tempered at 100 °C had the lowest fatigue resistance due to the presence of hard martensite, and the steel tempered at 250 °C showed the best results in terms of fatigue [8]. The fatigue resistance of heat-treated spring steel 60Si2CrVAT was studied in article [9] where the authors analyze the microstructure and fatigue fracture surface via the method of scanning electron microscopy.

Comparative investigations were performed to evaluate the effects of heat treatment on the microstructure material and resistance to fatigue. A review of the literature indicates various ways to increase fatigue resistance by performing different types of heat and thermochemical treatment.

It is known that heat treatments produce, on the surface of steel, parts of layers with high hardness stressed at compression. This explains why surface hardening, nitriding, and nitrocarburization increase the fatigue strength of the parts.

Sometimes, changes in the chemical components in the microstructure of the material at the surface layer can adversely affect fatigue resistance. A higher nitrocarburizing temperature decreases the hardness of the specimen surface and the fatigue resistance.

Some studies focus on the effect of post-heating treatment and residual stress on fatigue resistance. Thus, Chola et al. performed a thorough microstructural and fractographic analysis to evaluate the impact of the main factors which influence fatigue resistance. They used two groups of samples in the study: “polished” and “polished and polished”. The results showed that the fatigue strength of the polished samples was improved by heat treatment stress reduction [10].

The carbide layer on the surface of nitrocarburized chromium steels significantly increases hardness and wear resistance. However, due to its high fragility, it reduces fatigue strength. Moreover, the best fatigue characteristics are reached for nitrocarburized layers, the carbon content of which is about 0.7%. An increase in the carbon content above this value leads to a decrease in the cyclic strength of nitrocarburized steel [11].

Similar observations are presented in [12], where it was concluded that the higher the temperature, the lower the fatigue resistance. A good balance between ductility and strength derives from the transformation of martensite induced by deformation (DIMT). To prevent structural defects under static loads, high-performance alloys are designed with the formation-induced martensitic transformation method, as explained in paper [13], revealing the dual role of DIMT in increasing fatigue cracks through in situ observations.

The relationship between local stress and the local strength of the material is essential for the position of the potential fatigue crack. In [14], the authors detail the size of the crack and the extent of fatigue damage by making observations on its initiation and growth.

## 3. Materials and Devices

**Figure 1:**
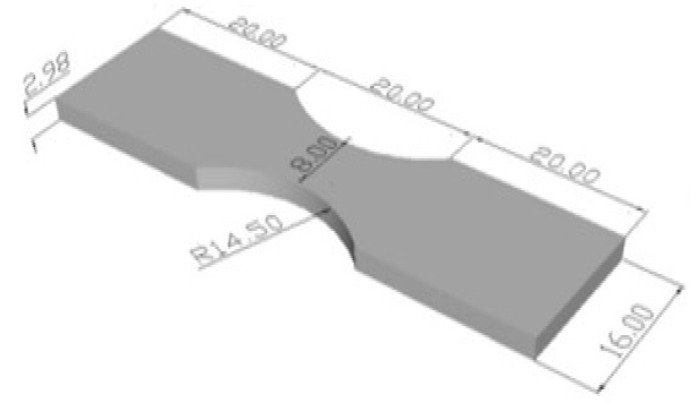
Size of the specimen used. Shape and dimension (in mm) of fatigue specimens [mm].

The specimens were prepared according to the above-mentioned standard for fatigue testing [2]. The geometry of the rectangular specimen with a size of 60 × 16 × 2.98 mm subjected to fatigue testing is presented in Figure 1. Experimental fatigue testing was performed at Russenberger Prüfmaschinen AG, Switzerland, using the Cracktronik machine at ambient temperature, and the Rumul soft expert software.

The stress amplitudes for nitrocarburizing-treated C75 steel in gas were 754, 724, 694, 664, 634, 604, 574, 544, 514, and 484 MPa; 812, 782, 752, 722, 692, 662, 632, 602, 572, and 542 MPa for nitrocarburizing-treated C75 steel in the salt bath; 711, 681, 651, 621, 591, 561, 531, 501, 471, and 441 MPa for heat-treated C75 steel in gas; and 767, 737, 707, 677, 647, 617, 587, 557, 527, 497 MPa for heat-treated C75 steel in the salt bath.

The differences in values are explained by the fact that we started from the value σ_adm_ and, with the relation [1], we determined the amplitudes. In the situation where we had four different types of treatments, it is obvious that we would have four different values for σ_adm_. This explains the different starting values. The rest of the values followed the recommendation of the standard to make the tests with a gradual decrease in amplitude from 30 MPa to 30 MPa.

σ_adm_ was the maximum stress introduced into the specimen in the section to be fractured. The specimens mounted on the Cractronik fatigue testing machine were subjected to an alternating symmetrical stress cycle, being loaded with maximum stress σadm.

The specimen tests were made in several steps of maximum tension. The specimens were loaded with descending loads. The intervals between steps were 30 MPa.

The C75S (1.1248) steel has a chemical composition presented in Table 1.

Crystallographic analysis was carried out at Oxford Instrument Wiesbaden using the EBSD (electron backscatter diffraction detector) system, to which a complementary sensor CMOS metal oxide semiconductor was attached.

Micro-hardness measurements were performed, starting from the outside to the inside, with a load of 0.5 Kgf for each gas and salt thermochemically treated specimen. The depth of the nitration case was determined by the distance from the surface to the point where the micro-hardness, determined according to DIN 50190 Part 3, was greater by a minimum of 50 HV than core hardness. Micro-hardness measurements were performed using a KB micro-hardness meter at the Härterei Reese laboratory in Brackenheim, Germany.

## 4. Experiment

The work to be completed and an experimentation plan were established in a logical succession of steps, as presented in Table 2. The investigations were carried out in several enterprises and research institutions. Following these experiments, the dependence between the heat treatments applied and the fatigue test parameters was highlighted.

Although, for C75 steel, if we are guided by its carbon content, nitrocarburization is not recommended, it was chosen as a solution to improve the fatigue resistance because it produces a high surface hardness by dissolving the base mass of precipitation of nitrogen and hard nitrides. The choice was based on a comparison with carburizing because it is performed at a higher temperature which produces a deeper oxidation of the surface of the part and an increase in austenite granules which degrades the mechanical properties, and implicitly those of fatigue. In addition, the oil-cooled parts have a risk of dimensional instability compared to the nitrocarburized parts where the nitriding temperature is below point A1 (from the Fe-C diagram), namely, 530–560 °C. Table 3 shows the conditions of the treatments applied.

**Figure 2:**
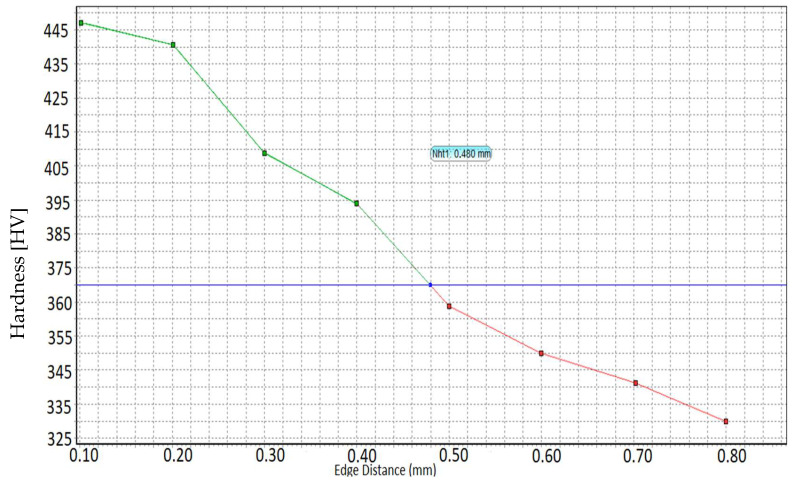
Diagram of gas-nitrocarburized layer depth. The green line in the diagram shows distance from the surface to the point that a limit hardness GH = (core hardness +50 HV); The blue line in the diagram shows the limit hardness GH; The red line shows core of the sample.

The thermochemical treatment of gas nitrocarburization was performed at Reese Brackenheim. The specimens were treated together with normal production batches in a Rubig oven at a temperature of 560 °C for 6 h. The measurements of the hardness, the thickness of the white layer, and the depth of the nitride layer are given in Table 4, and the depth of the nitrocarburized layer in the gas is represented in Figure 2.

**Figure 3:**
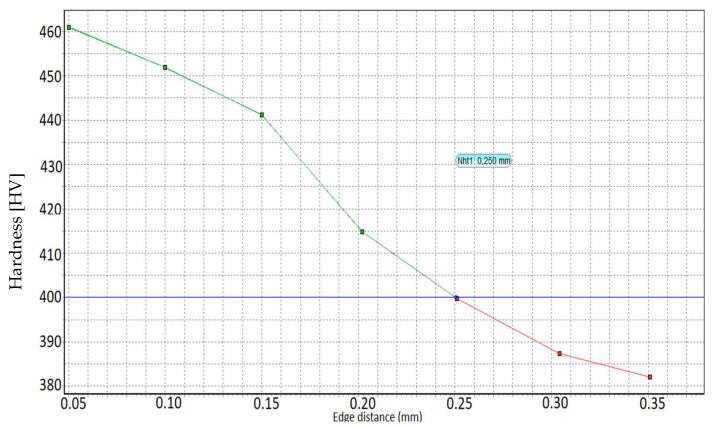
Diagram of salt bath-nitrocarburized layer depth. The green line in the diagram shows distance from the surface to the point that a limit hardness GH = (core hardness +50 HV); The blue line in the diagram shows the limit hardness GH; The red line shows core of the sample.

Nitrocarburization in the salt bath was performed at Durferrit Mannheim. The specimens were individually treated in an electrically heated salt bath oven TYP AS 140 at 530 °C for 3 h preheated at 350 °C. Measurements of hardness, white layer thickness, and depth of the nitrocarburized layer are given in Table 5, and the depth of the nitrocarburized layer in the salt bath is shown in Figure 3. It can be concluded that the greater the hardness of the core, the smaller the depth of the nitration case

Table 6 shows the difference between the temperature used and the surface hardness, as well as the depth of the nitration case.

The improvement in the gaseous environment was carried out within the Reese Härterei Brackenheim company. The specimens were heat-treated in a gas oven with a protective atmosphere. In Table 7, the parameters of the technological process for the heat treatment of gas tempering and quenching are shown: tempering at a temperature of 840 °C with a holding time of 3 h and cooling in oil, after which a hardness of 63 HRC was obtained. The tempering took place at a high temperature of 540 °C with a holding time of 2 h with cooling in the air, after which a hardness of 34 HRC was obtained. The structure obtained was a fine soorbite. 

Salt bath quenching and tempering heat treatment were performed at Durferrit-Mannheim Germany. Table 8 shows the heat treatment parameters to which the specimens were subjected.

After the heat treatment of gas and salt bath quenching and tempering, and after the thermochemical treatment of gas and salt bath nitrocarburizing, the fatigue resistance behavior of the specimens was determined experimentally. It has been shown that crack initiation can have different moments depending on the heat or thermochemical treatment applied to the material.

The specimens mounted on the Cractronik fatigue testing machine were subjected to an alternating symmetrical stress cycle loaded with maximum stress *σ*_adm_.

The amplitude with which the specimen was loaded was given by the bending moment which was calculated according to the formula [15]:

(1) $$M=\frac{b^{2\;}\cdot h}{6}\cdot \sigma_{adm}$$

where *M* is the bending moment, *σ*_adm_ is the maximum stress introduced into the specimen in the section to be fractured, *b* = 8 mm (measured on each specimen), and is the width of the calibrated portion, *h* = 2.98 mm (measured on each specimen), and is the thickness of the specimen. The fatigue tests on the salt bath nitrocarburizing specimens showed that the strength was much higher compared to the gas nitrocarburization specimens.

Following the two heat treatments applied in the gas and the salt bath in the case of tempering and quenching, it appears that the heat-treated sample in the gas had a high resistance to breaking due to fatigue with several cycles up to the breaking point of 17,403. This sample was subjected to a detailed investigation representing the macro-structural evolution carried out with the help of SEM (scanning electron microscope) analysis from the endowment of the fractography laboratory at the Institute for Research and Testing of BAM, Berlin.

Measurements were also made on the length of the fatigue rupture cracks. The results are shown in Table 9.

**Figure 4:**
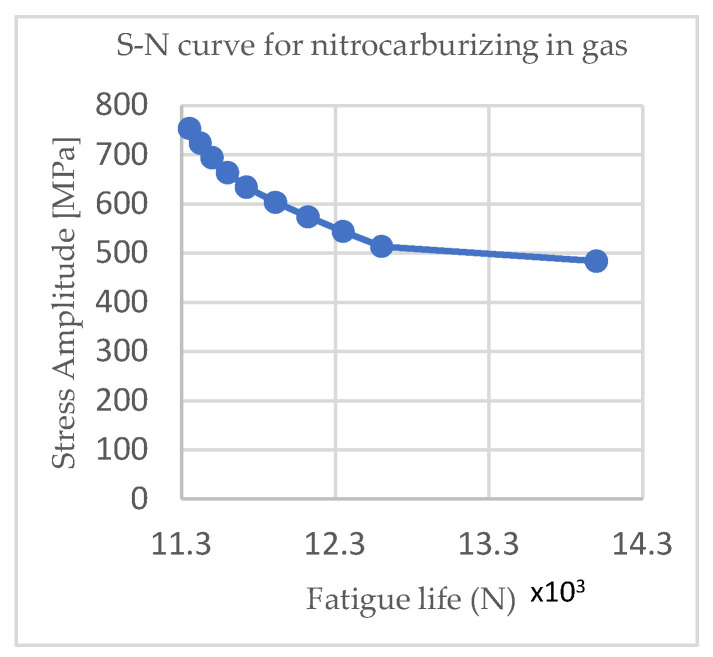
S–N curves for nitrocarburizing in gas.

**Figure 5:**
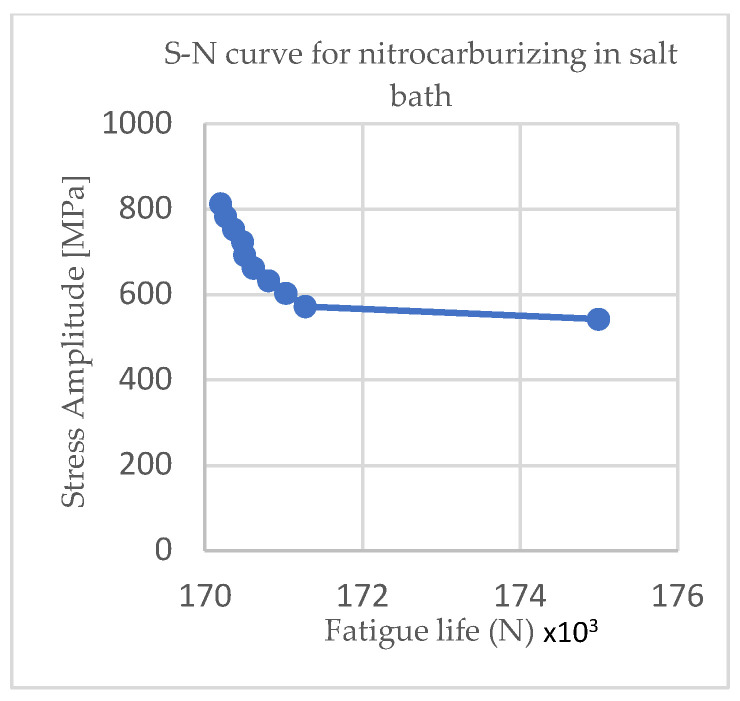
S–N curve for nitrocarburizing in the salt bath.

**Figure 6:**
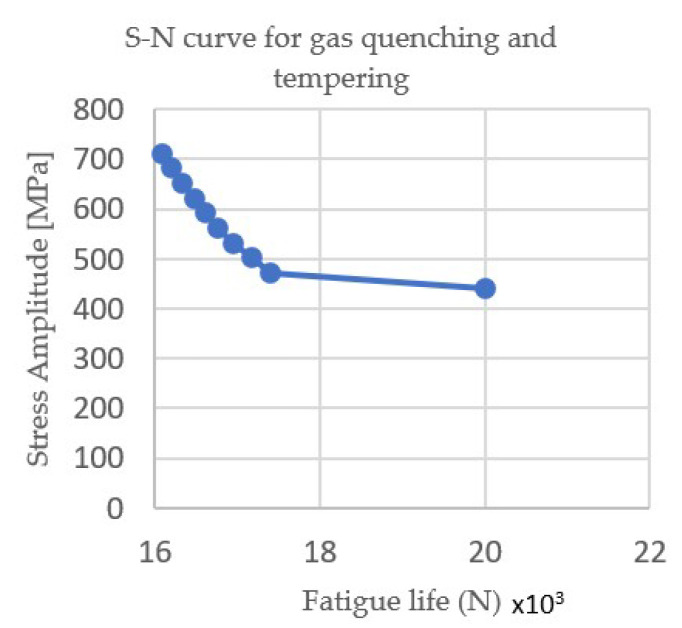
S–N curve for gas quenching and tempering.

**Figure 7:**
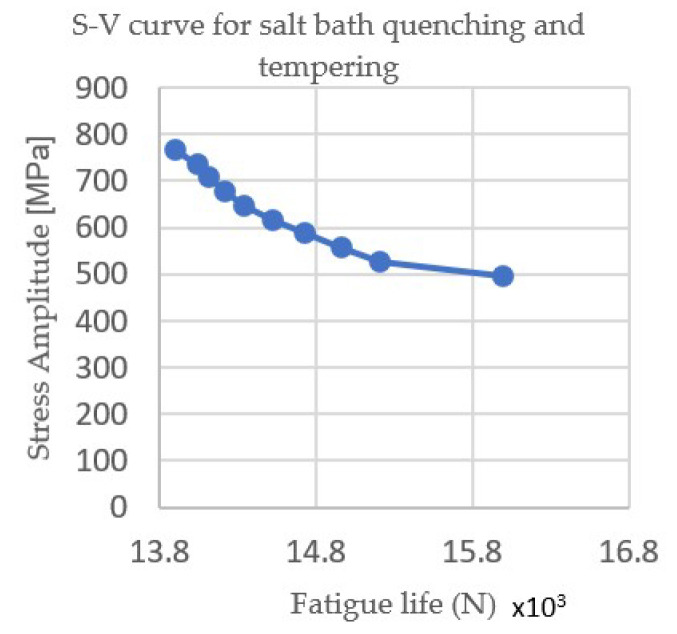
S–N curve for salt bath quenching and tempering.

Figure 4 and Figure 5 show the Wöhler curves for the nitrocarburizing samples in the gas and salt baths, respectively, and Figure 6 and Figure 7 show the Wöhler curves for the quenching and tempering gas samples.

From Figure 4, Figure 5, Figure 6 and Figure 7 it can be seen that as the cycle amplitude decreased, the resistance to fatigue increased. Thus, in Figure 4 and Figure 5, it can be seen that the fatigue life of the thermochemically treated specimens in the salt bath was significantly longer than those thermochemically treated in the gas due to changes in the microstructure that occurred through the diffusion of nitrogen and carbon.

Thus, in Figure 6 and Figure 7, it can be seen that the fatigue life of the heat-treated samples in the gas was significantly longer than those of the heat-treated in the salt bath due to changes in the hardness of the core and the surface of the sample.

Fatigue testing on the salt bath-nitrocarburized specimens showed that the resistance was much higher compared to the gas-nitrocarburized specimens. Table 10 shows the results of the fatigue testing.

Table 11 gives the treatment temperatures, hardnesses obtained, crack length, and the number of cycles to fracture for the gas and salt bath improved specimens.

Following the two heat treatments applied in the gas and salt bath, it appears that the gas heat-treated specimen had a high fracture strength with 17.403 cycles to fracture.

**Figure 8:**
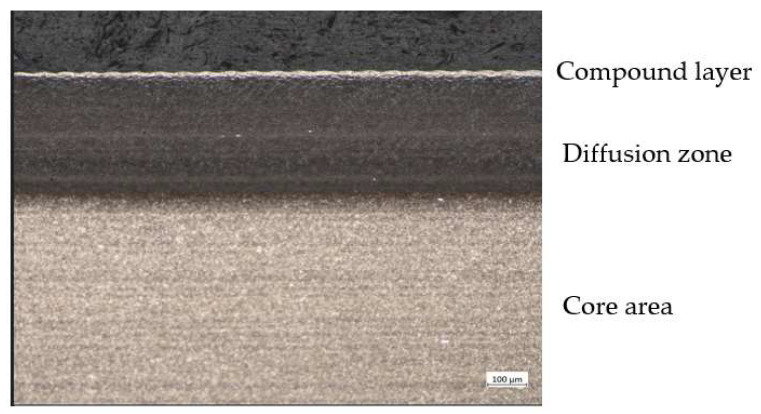
C 75 Stahl, nitrocarburized, etched with 3% Nital showing a dark etched diffusion zone with a compound layer and dark oxide layer.

After nitrocarburizing, the microstructure consisted of three zones: a compound layer at the surface, a diffusion zone, and an unaffected core area, as shown in Figure 8.

**Figure 9:**
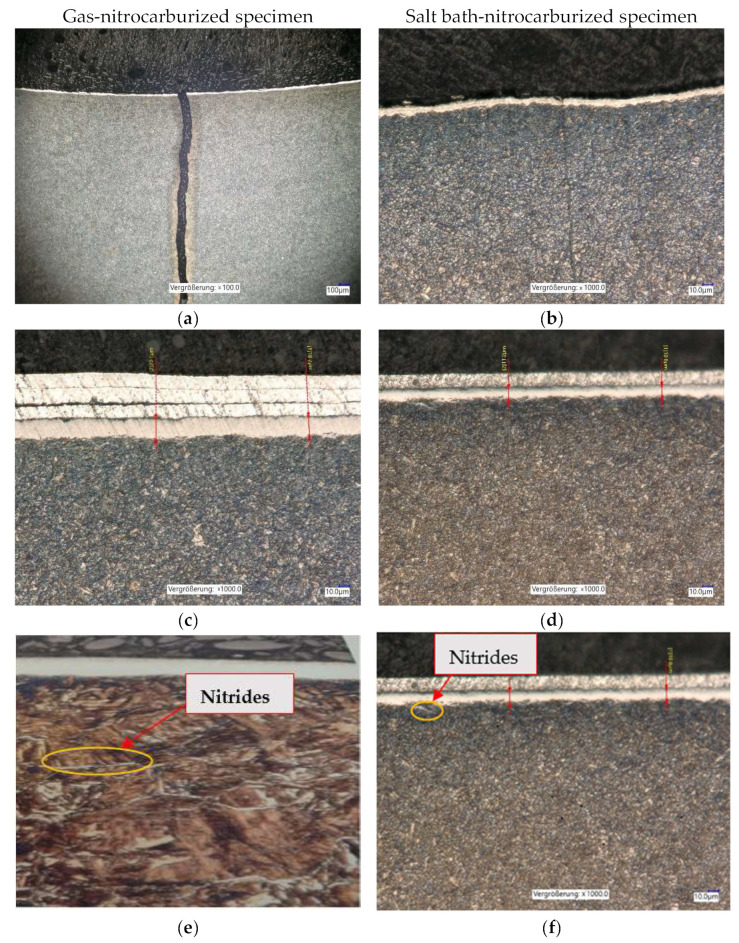
Metallographic analysis of gas- and salt bath-nitrocarburized specimens; (**a**) gas-nitrocarburized specimen, and microscopic crack control (Nital attack 3%); (**b**) salt bath-nitrocarburized specimen, microscopic crack control (Nital attack 3%); (**c**) gas-nitrocarburized specimen, microscopic control (×1000) with white layer thickness of 18.2–20.1 µm; (**d**) salt bath-nitrocarburized specimen, microscopic control (×1000) with white layer thickness of 10.8–11.2 µm; (**e**) nitride in the gas-nitrocarburized specimen, microscopic control (×1000); (**f**) nitride in the salt bath- nitrocarburized specimen, microscopic control (×1000).

**Figure 10:**
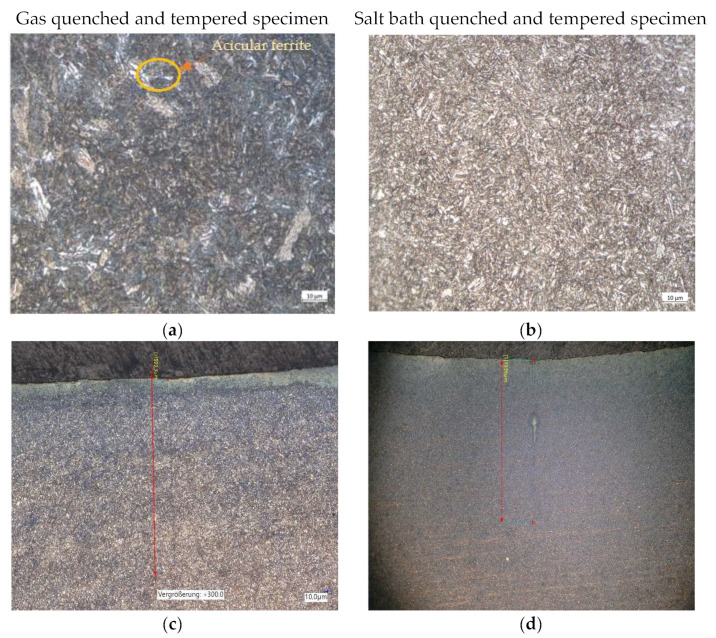
Microstructure obtained after gas and salt bath quenching and tempering; (**a**) microstructure—gas quenched and tempered specimen; (**b**) microstructure—salt bath quenched and tempered specimen; (**c**) determination of the crack length under the optical microscope (×300)—gas quenched and tempered specimen is 599.3 µm; (**d**) determination of the crack length under the optical microscope (×100)—salt bath quenched and tempered specimen is 1536 µm.

The microstructures resulting from gas and salt bath nitrocarburizing are shown in Figure 9 and Figure 10 for the gas and salt bath quenched and tempered specimens.

The presence of the difference between the results obtained from nitrocarburization in the gas and salt bath scenarios can be understood by studying the formation of the compound layer. The temperature, time, and the nitrogen and carbon potentials of the processing medium are important.

Figure 9a–d show the metallographic analyses for the heat and thermochemically treated specimens carried out with the Keyence microscope at ×100–1000 magnification. For the best possible protection of the white layer and to avoid its exfoliation during the preparation of the metallographic sample, the samples were wrapped in aluminum foil.

Figure 9a,b show that the crack was more predominant in the case of the gas-nitrocarburized specimens, which is explained by the depth of the diffusion layer of 0.48 mm compared to the 0.25 mm obtained in the salt bath.

Figure 9c shows the specimen thermochemically treated in gas at 560 °C for 6 h, compared with Figure 9d, where the specimen was thermochemically treated in a salt bath at 530 °C for 3 h. A difference in layer thickness can be seen between the gas-nitrocarburized specimens and salt bath-nitrocarburized ones. The thickness of the white layer was larger in the case of the gas-treated specimens in Figure 9c, where the white layer was 18.2–20.1 µm, compared to the salt bath-treated ones, where the white layer had a thickness of 10.8–11.2 µm Figure 9d.

Nitrides precipitate at the grain boundaries and they are characterized by very fine grains. With increasing temperature, the nitrides become thicker Figure 9e, and the duration of nitrocarburizing ensures the diffusion of nitrogen towards the core of the piece, influencing the depth of the nitration case. Due to the low cooling rate, only a few nitride needles were formed in the diffusion zone Figure 9f.

The white layer is found below the diffusion zone, in which nitrogen is stored in the iron lattice as a nitride precipitate which gives it a significant increase in hardness. [16].

The microstructural investigations presented in Figure 10a,b show the structure of the quenched and tempered material, respectively salt bath specimen what is it consisting of soorbite (acicular ferrite and globular carbides), which ensures a high fatigue resistance. Figure 10c,d show the microstructure of the specimens in the area where the length of the crack resulting from the fatigue testing is measured approximately.

To identify the cracks and crack lengths that appeared after fatigue testing, the specimens were subjected to fluorescent, Röntgen, and computed tomography analysis. With the help of the three analysis methods, we identified the critical area of the sample analyzed as the area where the crack appeared in the calibrated region b = 8 mm where the surfaces were subjected to stress and the stress concentration was at a maximum.

**Figure 11:**
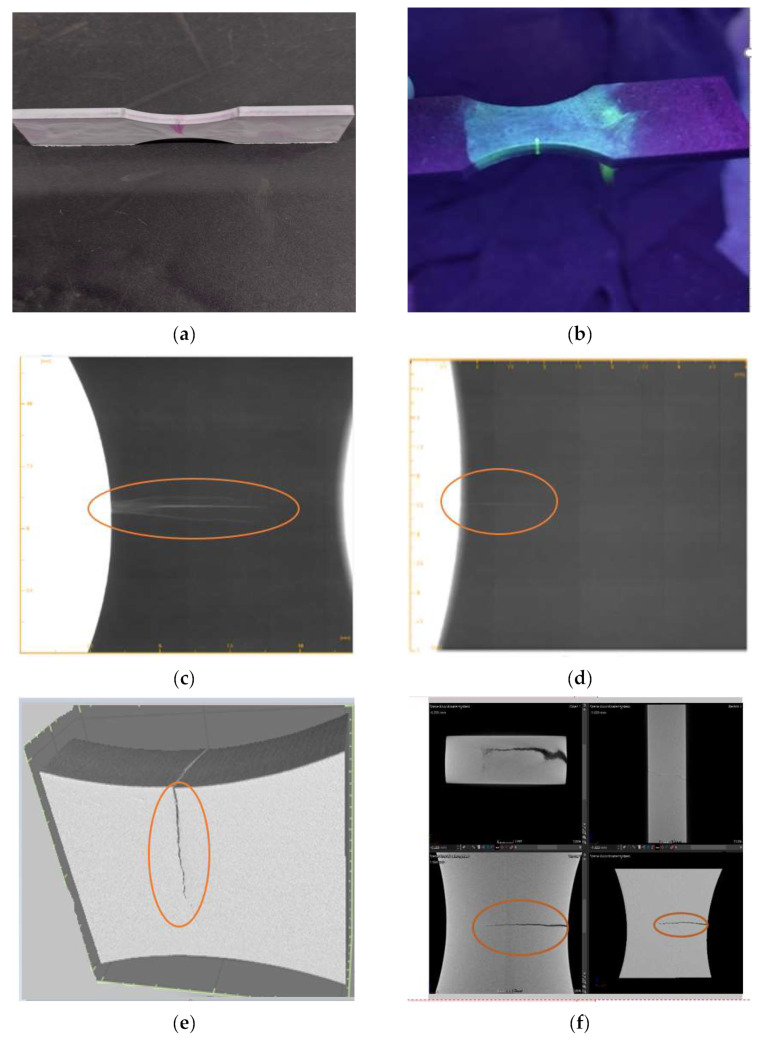
Non-destructive testing of gas- and salt bath-nitrocarburized specimens; (**a**) fluorescent analysis—gas-nitrocarburized specimen; (**b**) fluorescent analysis—salt bath-nitrocarburized specimen; (**c**) X-ray analysis—gas-nitrocarburized specimen; (**d**) X-ray analysis—salt bath-nitrocarburized specimen; (**e**) computed tomography—gas-nitrocarburized specimen with a crack length of 5.62 mm; (**f**), computed tomography—salt bath-nitrocarburized specimen with a crack length of 1.5 mm.

The penetrant inspection was used to detect surface-breaking discontinuities (e.g., cracks, pits, etc.) in the materials. With the penetrating liquids, the crack can be observed in the radius area in Figure 11a,b.

The radiographic examination shows us the interior of the sample and the areas in which their micro-cracks ordered material defects. For this purpose, the sample was positioned in a fully protective X-ray system and irradiated with electrically generated X-rays. The so-called live image was transmitted via a detector to a monitor, which was used by a trained X-ray inspector for evaluation.

With the help of the X-rays in Figure 11c,d, it was possible to determine the length of the crack. Through X-ray analysis, it can be seen that the crack was larger in the case of the nitrocarburized sample in the gas compared with the nitrocarburized one in the salt bath.

To determine exactly the length of the crack that appeared after the fatigue test, the same specimens were controlled with computed tomography. Analysis showed an average crack length of 1.5 mm in the case of the salt bath-nitrocarburized specimens Figure 11f, compared to 5.62 mm in the case of the gas-nitrocarburized specimens Figure 11e.

Following the two heat treatments of quenching and tempering applied to the gas and salt bath samples, it appears that the gas heat-treated specimen had a high fracture strength with 17,403 cycles to fracture. This specimen was subjected to a detailed investigation representing the macro-structural evolution carried out with SEM analysis from the fractography laboratory at the Institute for Materials Research and Testing BAM, Berlin. 

**Figure 12:**
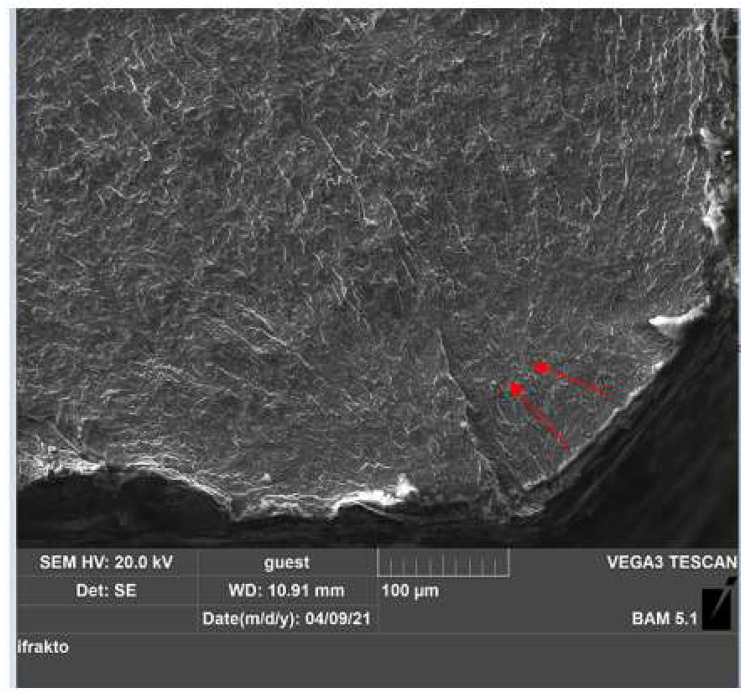
Macroscopic analysis for sample no. 1; fracture cracks on the fracture surface analyzed at a magnification of ×500.

**Figure 13:**
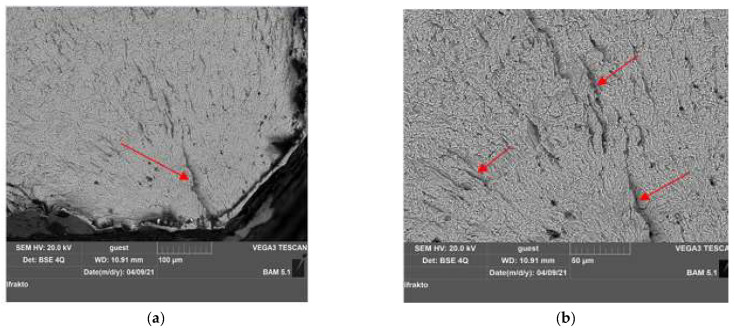
Macroscopic analysis for sample no. 1 C75 material: (**a**) crack initiation on the fracture surface analyzed at a magnification of ×500; (**b**) micro-cracks on the fracture surface analyzed at a magnification of ×1000.

Figure 12 and Figure 13 show SEM images of the fracture surface. We used magnification between 500× and 10.000× to analyze specimen no. 1, quenched in gas, and subjected to a maximum allowable stress of 712 MPa. Table 12 presents the testing characteristics of the specimen.

Figure 12 shows the fracture surfaces after the fatigue testing. The crack appeared in all samples on the surface of the tested specimen in the area of the calibrated portion b = 8 mm, the area where the surfaces were subjected to stress and where the stress concentration was maximum. From a macroscopic point of view, the appearance of two areas can be seen on the fracture surface of the specimen. Fracture paths and rest lines were macroscopically clearly recognizable as signs of fatigue fracture on fracture surfaces.

By increasing the magnitude of the SEM images in this area, the presence of unevenness with concentric arc arrangement to the place of initiation could be seen. This area was carefully analyzed to identify any material defects that led to the initiation of the fracture, and it was found that the crack initiated from the surface, developing transversely on the grains.

Figure 13 also presents cracks but at high magnification. On the fatigue fracture surfaces, the characteristic features such as the elliptical crack front, the indicated striations, and secondary cracks were documented.

In the longitudinal direction of the specimen, the main crack from the outside of the surface Figure 13a and the micro-cracks (striations in the material) that propagated in the same plane, having the same orientation inside the material, can be seen Figure 13b. This is because the specimen was subjected to high loading stress, namely, 24 Nm.

Fatigue cracks initiated at the level of the local slip bands tended to develop in the direction of maximum tangential stress. Due to the high amplitude used, it can be seen that the crack was transcrystalline (intra-granular) and developed transversely on the grains.

**Figure 14:**
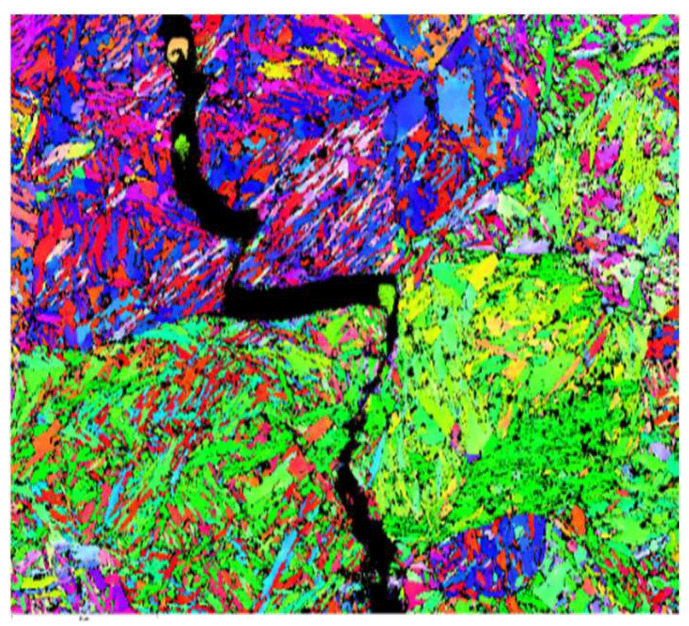
EBSD phase and orientation map of C75 specimens nitrocarburized in gas for 6 h at 560 °C, development of slip bands, and transcrystalline micro-cracks in the gas-nitrocarburized specimen (Rm = 1160 MPa).

In Figure 14, colors are mapped according to orientations in the standard stereographic.

## 5. Conclusions

In this work, the influence of thermal and thermochemical treatment parameters on the fatigue resistance of C75 steel was studied. It was found that the samples nitrocarburized in the salt bath presented a high fatigue resistance compared to the samples nitrocarburized in gas and the sample quenching and tempering in gas showed higher fatigue resistance compared to the quenching and tempering in a salt bath. Following the two heat treatments applied in the gas and salt bath, it appears that the gas-heat-treated specimen had a high fracture strength with 17,403 cycles to fracture.

1. The results of the study on thermochemically treated C75 steel specimens show that the fatigue resistance was improved depending on the temperature and the nitriding time used. The increase in fatigue resistance was determined by the diffusion zone of the nitrocarburized layer and by the nitride area. The fatigue limit for thermochemically treated specimens by nitrocarburizing increased by 94.3% compared to heat-treated specimens by quenching and tempering.

Comparing the gas-nitrocarburized specimen with a core hardness of 320 HV 10 with the salt bath-nitrocarburized specimen with a core hardness of 348 HV 10, it can be seen that the depth of the nitration case was greater in the case of the gas nitride specimen (Nht: 0.48 mm; Figure 2).

When setting the parameters of the thermochemical treatment, the temperature and time of exposure are very important because they have a tempering effect and they finally influence the depth of the layer. A higher nitrocarburizing temperature decreases the hardness of the specimen surface and vice versa. Table 6 shows the difference between the temperature used and the surface hardness, as well as the depth of the nitration case.

Fatigue testing on salt bath-nitrocarburized specimens shows that the resistance was much higher compared to gas-nitrocarburized specimens.

From the experiments, it can be concluded that sample C75 nitrocarburized in the salt bath with a small diffusion area of Nht: 0.25 mm but a high surface hardness of 600 HV 1 led to increased fatigue resistance with 170,200 cycles compared to the C75 sample nitrocarburized in gas with a large diffusion layer of Nht: 0.48 mm but a small surface hardness of 480 HV 1, leading to increased fatigue resistance with 11,352 cycles.

2. The specimens nitrocarburized in a salt bath at 530 °C for 3 h, when a depth of nitration of 0.25 mm and a surface hardness of 600 HV was obtained, had the highest fatigue limit compared to the specimens nitrocarburized in gas at 560 °C for 6 h, with a depth of nitration of 0.48 mm. It can be seen that the hardness of the surface and the core decreased with increasing temperature. It was found that a large depth of the diffusion zone was not necessary to increase fatigue resistance. The formation of carbonitrides in the surface layer led to increased fatigue resistance.

3. Although it involved higher costs, the thermochemical treatment in the salt bath was fully justified for C75 steel because it provided the highest increase in fatigue resistance by approx. 14 times compared to the gas thermochemical treatment, 9.8 times compared to gas quenching and tempering, and by 11.2 times compared to salt bath quenching and tempering. 

4. The improvement in fatigue resistance in the case of the thermochemically treated specimens was related to the increase in surface hardness (600 HV 1), the formation of compressive residual stress in the surface layer, and finally the carbonitrides formed in the layer.

5. The thermochemical treatment of nitrocarburizing increased the micro-hardness in the diffusion layer. When setting the parameters of the thermochemical treatment, the temperature and the time of exposure are very important because they have a tempering effect and they influence the depth of the layer. A higher nitrocarburizing temperature decreased the hardness of the specimen surface and fatigue resistance.

6. It can be concluded that the propagation of the crack is determined by the type of heat treatment chosen, the parameters obtained from it, and the stress introduced into the specimen.

7. The critical area of the analyzed specimen was identified in the calibrated region b = 8 mm, where the surfaces were subjected to stresses and the stress concentration was maximum. It can be seen that the nanostructure of the material was destroyed with increasing amplitude, with micro-cracks destroying the crystalline lattice. Each crystalline grain had its orientation at the crystallographic level. The high-amplitude room temperature fatigue testing showed that the crack passed through the crystal (intragranular crack).

## Figures and Tables

**Table 1:** Chemical composition of the material.

	Material C75S (1.1248)	Chemical Composition (%)					
		C	Si	Mn	Cr	Mo	Ni
	Standard	0.70–0.80	0.15–0.35	0.60–0.90	0.10–0.40	Max 0.10	Max 0.20
0.	Material analysis Fa. Zelos Zerspannung	0.72	0.22	0.60	0.19	-	-
1.	Material analysis Fa. Nobitschek	0.75	0.23	0.68	0.15	-	-

NOTE: C75 steel was purchased in two different states: 0. Quenched and tempered, with a hardness of 1381–1385 MPa (B2), from Zelos Zerspanung-Bessenbach, Germany. This steel was nitrocarburized in a gas and salt bath. 1. Annealed, with a hardness of 697–717 MPa, from Nobitschek GmbH, Iserlohn, Germany. This steel was quenched and tempered in a gas and salt bath.

**Table 2:** The planning of the experiment.

Nr. Crt	Stage	Targeted Results	Type of Testing	Place of Measurement
1	Check the chemical composition of material	Composition of the material	Spectrometer	Reese Härterei
2	Sampling	60 × 16 × 2.98 mm	Turning machine	Zelos Zespanung
3	Identification of specimens	Laser marking	Laser	Fa. Honecker GmbH and Fa. Mathias Meidlinger Brackenheim
4	Heat and thermochemical treatment	Gas quenching and tempering Gas nitrocarburizing Salt bath quenching and tempering Salt bath nitrocarburizing	Gas furnace Gas furnace Salt bath Salt bath	Härterei Reese Brackenheim Härterei Reese Brackenheim Durferrit Mannheim Durferrit Mannheim
5	Fatigue testing	Number of cycles to fracture	Rumul Cractronick	Russenberger Prüfmaschinen AG, Schwizerland
6	Non-destructive testing of nitrocarburized specimens	Identification of cracks with penetrating liquid Crack identification with X-Ray analysis Crack length measurement by computer tomography	Spray/X-Ray devices Computer tomography	BMB Gesellschaft für Materialprüfung-Bad Rappenau
7	Metallography of nitrocarburized specimens	Determination of microstructure	Keyence Microscope	Reese Härterei Brackenheim
8	Conclusions on nitrocarburizing	Obtained results	Micro-hardness meter KB 30S	Reese Härterei Brackenheim
9	Non-destructive testing of quenched and tempered specimens	Identification of cracks with penetrating liquids Crack identification with X-Ray analysis Crack length measurement with computer tomography	Spray/X-Ray devices Computer tomography	BMB Gesellschaft für Materialprüfung-Bad Rappenau
10	Metallography of quenched and tempered specimens	Microscopic determination	Keyence Microscope	Reese Härterei Brackenheim
11	Macro-structural analysis	Determination of macro-structure	Electron microscope	Fractography Laboratory of the Institute for Materials Research and Testing BAM, Berlin
12	Crystallographic analysis	Lattice deformation, crack propagation mode	EBSD (electron backscatter diffraction detector)	Oxford Instruments Wiesbaden

**Table 3:** Heat and thermochemical treatment conditions.

**C75 Material**	**Heat/** **Thermochemical** **Treatment**	**Preheating (°C)**	**Duration (min)**	**Temp.** **(°C)**	**Duration (min)**	**Stress** **Relief (°C)**	**Temp. (°)**	**Rm (MPa)**	**NHD (mm)**	**White Layer** **(µm)**	**Core** **Hardness** **(HV 10)**
	**Gas nitrocarburizing**	-	-	560	360	-	-	1160	0.48	18.2–20.1	320
	**Salt bath nitrocarburizing**	350	30	530	180	-	-	1250	0.25	10.8–11.2	348
	**Gas quenching and tempering**	-	-	840	180	-	540	1095	-	-	-
	**Salt bath quenching and tempering**	350	30	840	15	Salt AS 140 180 °C/1 h	Salt AS 140 500 °C/1 h	1180	-	-	-

**Table 4:** Results of gas nitrocarburizing treatment.

The Hardness of the Specimen Subjected to Gas Heat Treatment					Depth of Layer	The Thickness of the White Layer
Surface	Core		Intermediate Area		(mm)	(µm)
(HV 1)	(HV 10)	(MPa)	(HRC)	(MPa)		
480	320	1030	37	1160	0.48	18.2–20.1

**Table 5:** Results of salt bath nitrocarburizing treatment.

The Hardness of the Specimen Subjected to Salt Bath Heat Treatment					Depth of Layer	The Thickness of the White Layer
Surface	Core		Intermediate Area		(mm)	(µm)
(HV 1)	(HV 10)	(MPa)	(HRC)	(MPa)		
600	348	1125	39.5	1250	0.25	10.8–11.2

**Table 6:** Parameters of nitrocarburizing.

Nitrocarburizing	Temperature (°C)	Time of Exposure (h)	Surface Hardness(HV1)	Core Hardness (HV 10)	Layer Depth (mm)
In salt bath	530	3	600	350	0.25
In gas	560	6	480	320	0.48

**Table 7:** Parameters of gas quenching and tempering heat treatment.

**C75**	**Applied** **Treatment**	**Quenching** **(°C)**	**Time of** **Exposure** **(h)**	**Cooling** **Medium**	**High** **Tempering** **(°C)**	**Time of** **Exposure** **(h)**	**Cooling Medium**	**Hardness** **(HRC)**	**Rm** **(MPa)**
	Gas quenching and tempering	840	3 h	oil	540	2 h	air	33.68–35.49	1095

**Table 8:** Parameters of salt bath quenching and tempering heat treatment.

**C75**	**Applied** **Treatment**	**Preheating Temperature (°C)**	**Preheating** **Duration (min)**	**Quenching** **Temperature (°C)**	**Quenching Medium**	**Quenching Duration (min)**	**Stress Relief (°C)**	**Stress Relief Duration (min)**	**Stress Relief Medium**	**Tempering Temperature (°C)**	**Tempering Medium**	**Tempering Duration (min)**	**Rm (MPa)**
	Salt bath quenching and tempering	350	30	840	Salt GS 540/R2	15	180	60	Salt AS 140	500	Salt AS 140	60	1180

**Table 9:** Heat treatment process and crack length.

Quenching and Tempering in Salt Bath and Gas	Crack Length (Measured with an Optical Microscope) (mm)
In salt bath	1.526
In gas	0.828

**Table 10:** Fatigue resistance after thermochemical treatment.

Nitrocarburizing	Rm (MPa)	No. of Cycles (N)	Crack Size (mm)	Not (mm)	Core Hardness (HV 10)	Surface Hardness (HV 1)	White Layer (µm)	Temperature (°C)	Duration (h)
In gas	1160	11,352	5.62	0.48	320	480	18.2–20	560	6
In salt bath	1250	170,200	1.5	0.25	350	600	10.8–11.2	530	3

**Table 11:** Fatigue resistance after thermal treatment.

Quenching and Tempering in Salt Bath and Gas	Quenching Temperature (°C)	Tempering Temperature (°C)	Rm (MPa)	No. of Cycles (N)	Crack Size (mm)
In salt bath	840	500	1180	15,216	1.526
In gas	840	540	1095	17,403	0.828

**Table 12:** Testing characteristics.

Material	Stress Step	Rm (MPa)	∂_adm_ (MPa)	Amplitude (Nm)	Lifetime (No. of Cycles)
C75	Step 1	1095	σ_adm_ = Rm × 0.65	24	17,403

## Data Availability

(1). RUSSENBERGER PRÜFMASCHINEN AG, https://www.rumul.ch/index.php (accessed on 23 June 2022); (2). REESE HÄRTEREI BRACKENHEIM DEUTSCHLAND, https://www.haerterei.com/ (accessed on 23 June 2022); (3). DURFERRIT GmbH MANNHEIM De, https://www.nitriersalze.com/ (accessed on 23 June 2022); (4). BAM-Federal Institute for Materials Research and Testing (Bundesanstalt für Materialforschung und -prüfung) Deutschland, https://www.bam.de/Navigation/DE/Home/home.html (accessed on 23 June 2022).
